# Dysregulation of sterol regulatory element-binding protein 2 gene in HIV treatment-experienced individuals

**DOI:** 10.1371/journal.pone.0226573

**Published:** 2019-12-17

**Authors:** Anuoluwapo Sopeyin, Lei Zhou, Min Li, Lydia Barakat, Elijah Paintsil

**Affiliations:** 1 Department of Pediatrics, Yale University School of Medicine, New Haven, Connecticut, United States of America; 2 Department of Medicine, Yale University School of Medicine, New Haven, Connecticut, United States of America; 3 Department of Pharmacology, Yale University School of Medicine, New Haven, Connecticut, United States of America; 4 School of Public Health, Yale University, New Haven, Connecticut, United States of America; Beijing Key Laboratory of Diabetes Prevention and Research, CHINA

## Abstract

Although antiretroviral therapy (ART) has resulted in a marked decrease in AIDS-related morbidity and mortality, the therapeutic benefit is often limited by side effects such as metabolic derangement such as lipodystrophy and hyperlipidemia and cardiovascular diseases. These side effects are pervasive in people living with HIV (PLWH). However, the underlying mechanisms are not completely understood. We investigated the effects of ART on cholesterol biosynthesis genes. This is a retrospective analysis of data and specimens collected during a cross-sectional, case-control study of ART-induced toxicity. Cases were HIV treatment-experienced individuals with HIV viral suppression and no diagnosis of ART-associated toxicity (n = 18), and controls were HIV-uninfected individuals (n = 18). The mRNA expressions of 3-hydroxy-3-methylglutaryl-coenzyme A reductase (HMGCR) and ATP binding cassette transporter A1 (ABCA1) were significantly upregulated in cases (HIV+) compared to controls (HIV-), as well as the corresponding protein expression level of HMGCR. We observed dysregulation between sterol regulatory element-binding protein 2 (SREBP-2, sensory control) and HMGCR and low-density lipoprotein receptor (LDLR) pathways. Dysregulation of cholesterol biosynthesis genes may predate clinical manifestation of ART-induced lipid abnormalities.

## Introduction

Antiretroviral therapy (ART)-associated metabolic derangement and metabolic syndrome (MetS) are more prevalent than ART-associated toxicities such as lactic acidosis, peripheral neuropathies, cardiomyopathies, and pancytopenia [1–5]. In adults, MetS is defined as having at least three out of five of the following components: impaired fasting glucose or diabetes, hypertension, central obesity (increased waist circumference), elevated triglycerides or reduced high-density lipoprotein (HDL) cholesterol [6]. The prevalence of MetS in people living with HIV (PLWH) is as high as 83%, particularly in PLWH on protease inhibitors (PI)-based regimens [7], compared to 34% in the general population [8]. MetS has been associated with an increased risk of cardiovascular diseases (CVDs) such as myocardial infarction (MI), atherosclerosis, and stroke [9, 10].

The high prevalence of MetS and CVDs in PLWH may be due to a complex interplay of HIV infection [11, 12], ART exposure, other viral co-infections [13, 14], and traditional risk factors such as genetic predisposition genetics [15] and lifestyle habits. However, the underlying mechanisms are not well known. We recently observed that CEM cells exposed to 1x- and 4x-C_max_ of various antiretroviral combinations resulted in differential expressions of 122 out of 48,226 genes using microarray analysis (published [16] and unpublished data). Over a third of those genes belonged to the cholesterol biosynthesis pathway. Based on our findings, we hypothesized that ART could perturb cholesterol biosynthesis genes before manifestation of overt signs and symptoms of lipid abnormalities and MetS. We investigated the effect of ART on cholesterol biosynthesis in peripheral blood mononuclear cells (PBMCs) of HIV treatment-experienced individuals (cases) compared to HIV-negative healthy individuals (controls).

We interrogated four major pathways genes involved in cholesterol regulation using mRNA and protein expression studies: sensory control (sensor sterol regulatory element binding protein 2, SREBP-2), de novo synthesis (3-hydroxy-3-methylglutaryl-coenzyme A reductase, HMGCR), cholesterol uptake (low-density lipoprotein receptor, LDLR), and efflux (ATP binding cassette transporter A1, ABCA1). We also measured the expression of AMP-activated protein kinase A1 & B2 (AMPK A1 & AMPK B2, precursors of the cholesterol synthesis pathway.

## Materials and methods

### Study participants and procedures

Study participants were enrolled at the Yale-New Haven Hospital from April 2011 to March 2013. The details of the study design for this cohort have been described previously [17]. In brief, for this cholesterol sub-study, cases comprised HIV-infected individuals on ART for at least 12 months without clinical and/or laboratory toxicities including MetS. Cases were matched by age, sex, and race/ethnicity to HIV-negative controls. All participants gave their written informed consent before participation in the study. The study protocol was approved by the Institutional Review Board of the Yale School of Medicine.

At study enrollment, participants answered a brief survey comprised of demographic characteristics and past medical history. Medical records of HIV-infected participants were reviewed, and disease characteristics and laboratory data (complete blood count, serum chemistries, liver function test, lipid profile, urinalysis, HIV RNA copy number, and CD4+ T-cell count) were extracted. Each participant gave about 20 ml of venous blood at the time of enrollment. Peripheral blood mononuclear cells (PBMCs) were isolated from whole blood within 2 hours of collection using Ficoll gradient (Ficoll-Hypaque; ICN) as described previously [18]. Aliquots of PBMCs were stored at -80°C until RNA extraction for cholesterol biosynthesis pathway gene expression experiments, and Western blot analysis. This sub-study included only study participants with sufficient archived PBMCs for the analysis (cases, n = 18, and controls, n = 18).

### RNA isolation and cholesterol biosynthesis gene expression assay

RNA was isolated from PBMCs using the TRIzol^®^ Reagent Kit according to the manufacturer’s instructions as previously described [19], after which quantitative real-time PCR (qRT-PCR) was performed for mRNA expressions of cholesterol biosynthesis genes (see Table 1 for primer sequences): SREBP-2, HMGCR, LDLR, ABCA1, AMPK A1 and AMPK B2. The housekeeping gene encoding glyceraldehyde 3-phosphate dehydrogenase (GAPDH) was used as an internal control for all reactions. Melting curve analysis was conducted on the qRT-PCR output to ensure that no false-positive results were included in the analysis. Data were obtained from at least two independent experiments with duplicates in each experiment. The fold change in gene expression was calculated as 2^-Δ*CT*^, where Δ*CT*_(case)_ = (*CT*_(gene of interest)_ − *CT*_(GAPDH)_), and Δ*CT*_(healthy control)_ = (*CT*_(gene of interest)_ − *CT*_(GAPDH)_).

**Table 1:** Primer sequence.

Gene Name	Gene ID	Reference Sequence	Forward Reverse	Product Size
Sterol regulatory element binding protein 2	SREBP2	NM_004599	5′-TGGCTTCTCTCCCTACTCCA-3′ 5′-GAGAGGCACAGGAAGGTGAG-3′	153
HMG coenzyme reductase A	HMGCR	NM_000859	5’-TTCGGTGGCCTCTAGTGAGA-3’ 5’-GATGGGAGGCCACAAAGAG-3’	99
Low-density lipoprotein receptor	LDLR	NM_000527	5'-GCTTGTCTGTCACCTGCAAA-3' 5′-AACTGCCGAGAGATGCACTT-3'	190
Adenosine triphosphate–binding cassette transporter A1	ABCA1	NM_005502	5'-AACAGTTTGTGGCCCTTTTG-3' 5'-AGTTCCAGGCTGGGGTACTT-3'	156
AMP Kinase A1	AMPK A1	NM_006251	5’-ACCTTCGGCAAAGTGAAGG-3’ 5’-CACATCAAGGCTCCGAATCT-3’	96
AMP Kinase B2	AMPK B2	NM_005399	5’-GTGTTCAGCCTCCCTGACTC-3’ 5’-CCTTCAGACCAGCGGATAAC-3’	125

### Western blot analysis of protein expression of cholesterol biosynthesis genes

Western blot analysis was performed as described previously [20] using total cell protein extracts from PBMCs. Measurements were conducted on participants with sufficient samples for western blot analysis (controls, n = 8; and cases, n = 8). Tubulin was used as the housekeeping gene. Primary antibodies: HMGCR and ABCA1 were used at 1:2000 (Abcam, Cambridge, MA); secondary antibodies were HRP conjugated anti-rabbit antibodies anti-mouse antibodies at 1:2000 and 1:5000, respectively (Cell Signaling Technology, Danvers, MA). Enhanced chemiluminescence substrate was used for signal development (PerkinElmer, Shelton, CT). Quantity One Analysis Software was used to quantify band density from the films.

### Statistical analysis

We report data as medians with 25^th^– 75^th^ percentile interquartile ranges (unless otherwise stated) and as frequencies with percentages for continuous and categorical variables, respectively. We used the Wilcoxon signed-rank test to compare continuous variables and a linear regression model to examine associations. P-values were considered significant if <0.05. All statistical analyses were performed using GraphPad Prism software.

## Results

### Characteristics of study participants

The demographic and clinical characteristics of study participants are illustrated in Table 2. The mean age was 53 years (range, 38 to 72 years), with 67% being males. The race/ethnicity comprised 28% non-Hispanic whites, 6% Hispanic white and 66% African Americans. The ART regimen of the cohort was mostly tenofovir/emtricitabine (33%) plus a protease inhibitor (PI) or an integrase inhibitor, tenofovir/emtricitabine/efavirenz (50%) and zidovudine/lamivudine (17%).

**Table 2:** Demographic and clinical characteristics of study participants.

Variable		HIV un-infected individuals (Controls, n = 18)	HIV infected individuals on antiretroviral therapy (Cases, n = 18)
Mean Age (Range), years		53 (38–72)	53 (38–72)
Gender	Male	12	12
	Female	6	6
Race	White non-Hispanic	5	5
	White Hispanic	1	1
	African American	12	12
Mean CD4 count (range) (count/μL)		N/A	735 (264–1159)
Mean Viral load (range) (copies/mL)		N/A	23 (20–79)
Mean Duration of exposure to treatment (range) (yrs)		N/A	4.77 (1–7.5)
Treatment Regimen (%)	NRTI	N/A	9
	NRTIs/NNRTI	N/A	9
Mean Cholesterol (range)		N/D	175 (72–248)
Mean HDL (range)		N/D	52 (25–82)
Mean LDL (range)		N/D	99 (9–158)
Mean Triglycerides (range)		N/D	129 (56–258)

N/A, not applicable

N/D, not determined for healthy volunteers

NRTI, Nucleoside Reverse Transcriptase Inhibitor

NNRTI, Non-nucleoside Reverse Transcriptase Inhibitor

### mRNA expression of cholesterol biosynthesis genes in study participants

**Fig 1:**
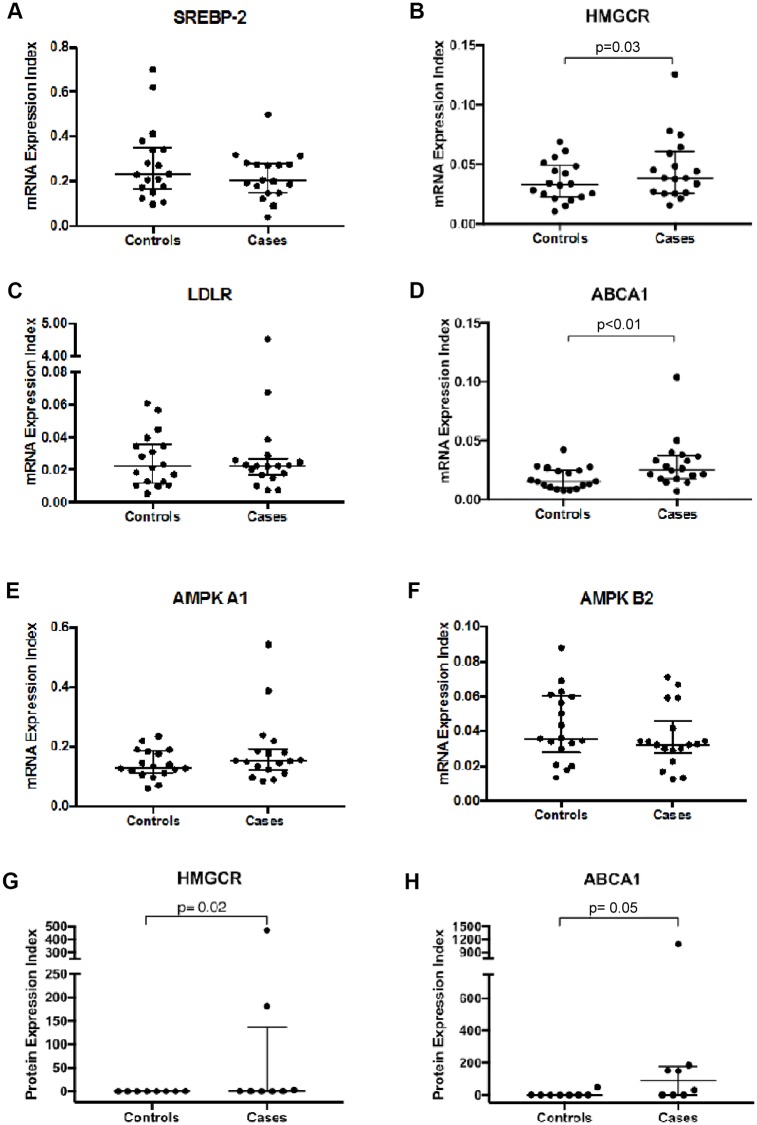
mRNA and protein expression of cholesterol biosynthesis genes in peripheral blood mononuclear cells (PBMCs) of participants. HIV positive individuals on ART (cases, n = 18) and HIV negative individuals (controls, n = 18). A. mRNA expression of Sterol response element binding protein 2 (SREBP-2). B. mRNA expression of HMG coenzyme reductase A (HMGCR). C. mRNA expression of Low-density lipoprotein receptor (LDLR). D. mRNA expression of Adenosine triphosphate–binding cassette transporter A1 (ABCA1). E. mRNA expression of AMP Kinase A1 (AMPK A1). F. mRNA expression of AMP Kinase A2 (AMPK A2). Protein expression of cholesterol biosynthesis genes in PBMCs of HIV positive individuals on ART (cases: N = 8) and HIV negative individuals (controls: N = 8). The density of the bands was quantified using Quantity One Analysis Software. Data are median (25^th^– 75^th^ percentiles of interquartile range), p values represent Wilcoxon matched-pairs signed-rank test, with significance being p < 0.05. G. protein expression of HMGCR. H. protein expression of ABCA1.

Elevated cholesterol levels are associated with foam cell development and an increased risk of cardiovascular events [21]. We quantified the mRNA expression of genes involved in cholesterol biosynthesis- SREBP-2, HMGCR, LDLR, and ABCA1 as well as genes involved in the signaling of cellular energy states- AMPK A1 and AMPK B2 using qRT-PCR. Samples from cases (treatment-experienced PLWH) and controls (individuals without HIV) were used in this quantification. HMGCR and ABCA1 mRNA expression levels were upregulated in cases compared to healthy controls (p = 0.03 and p<0.01, respectively) (Fig 1). There was no significant difference in SREBP-2 expression among cases and controls, although cases tended to have a lower expression of SREBP-2 and AMPK B2 compared to healthy controls. Similarly, there was no statistically significant difference in the expression of LDLR and AMPK A1 between cases and controls.

### Protein expression of cholesterol biosynthesis genes in study participants

**Fig 2:**
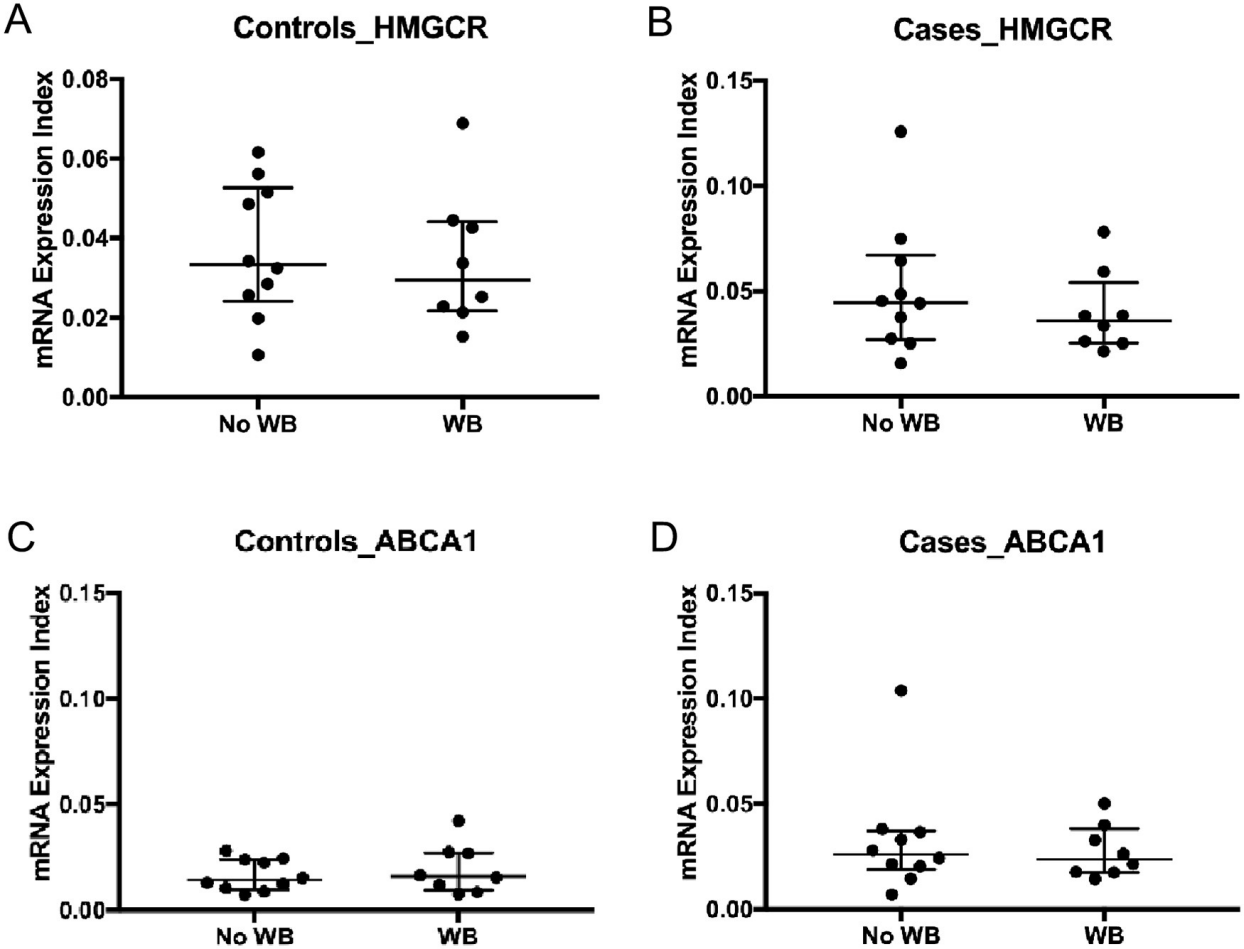
mRNA expression levels in patients with protein expression level results (western blots (WB)) compared to those without (No WB) to determine whether included participants were comparable to excluded participants. A. Controls- mRNA expression comparison of HMGCR. B. Cases- mRNA expression comparison of HMGCR. C. Controls- mRNA expression comparison of ABCA1. D. Cases- mRNA expression comparison of ABCA1. n = 10 (No WB-), n = 8 (WB). Data are median (25^th^– 75^th^ percentiles of interquartile range), p values represent Wilcoxon matched-pairs signed-rank test, with significance being p < 0.05.

With the significant increase in mRNA expressions of HMGCR and ABCA1 in cases, we investigated whether this translated to protein expressions. Western blot analysis was performed as described previously [20] using tubulin as the housekeeping gene. The Western blot analysis was conducted on 16 participants with sufficient samples (cases n = 8 and controls n = 8). We observed a corresponding increase in protein expression of HMGCR (p = 0.02), however the ABCA1 expression levels did not attain statistical significance (p = 0.05) in cases (Fig 1G and 1H). To exclude the possibility that the sub-group of patients with adequate cells whose protein expression levels were quantified represented a non-random sampling, we compared the mRNA expression levels of the patients with and without protein expression level results and found no statistically significant difference (Fig 2).

### Correlation of cholesterol biosynthesis genes in study participants

**Fig 3:**
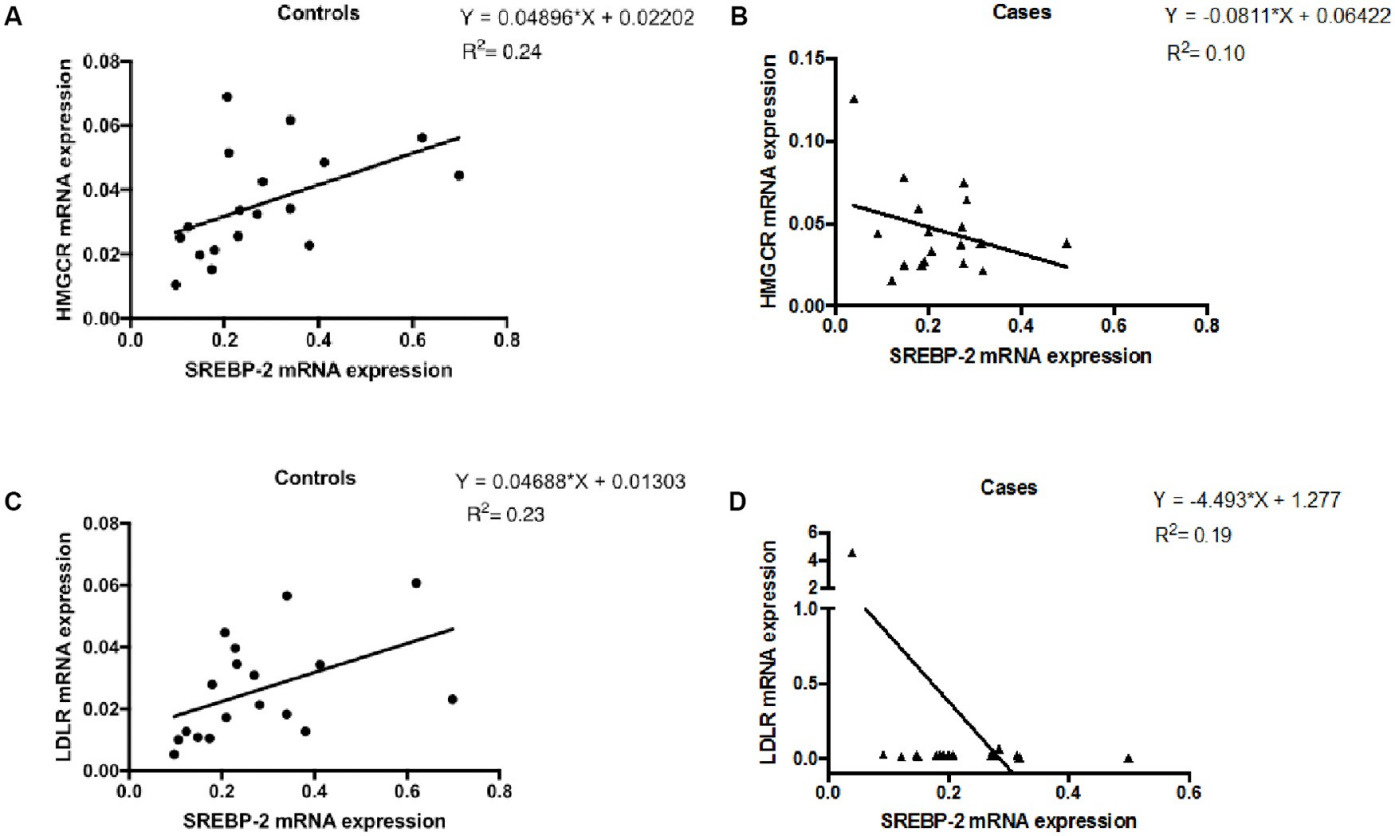
Correlation analysis of mRNA expression of cholesterol biosynthesis genes in peripheral blood mononuclear cells (PBMCs) of participants. HIV positive individuals on ART (cases, n = 18) and HIV negative individuals (controls, n = 18). Linear regression analysis was performed and significance was noted for p values < 0.05. R^2^ values are reported for correlations with significance. A. The mRNA expression of SREBP-2 vs. HMGCR in controls. B. The mRNA expression of SREBP-2 vs. HMGCR in cases. C. The mRNA expression of SREBP-2 vs. LDLR in controls. D. The mRNA expression of SREBP-2 vs. LDLR in cases.

In health, there is a positive correlation between SREBP-2 and HMGCR as well as SREBP-2 and LDLR [22, 23]. Therefore, with differential upregulation of HMGCR and ABCA1 in cases and controls, we investigated the correlation between SREBP-2 and HMGCR, and SREBP-2 and LDLR. As expected in controls (N = 18), there was a positive correlation between SREBP-2 and HMGCR (R^2^ = 0.24, p = 0.04), and LDLR (R^2^ = 0.23, p = 0.05) (Fig 2). To our surprise, the correlations among the cases were negative (Fig 3). Even when the outlier in Fig 3D was excluded, the data still reflected a negative correlation in cases (data not shown).

## Discussion

ART is associated with adverse effects and toxicities that can significantly decrease clinical efficacy [24], however, the underlying molecular mechanisms are under-studied. We measured the expression levels of genes involved in cholesterol biosynthesis and found an upregulation of HMGCR and ABCA1. There was corresponding increase in protein expressions of HMGCR. Correlation studies confirmed the previously documented relationship between the genes in healthy individuals. In health, there is a positive correlation between SREBP-2 and HMGCR as well as SREBP-2 and LDLR [22, 23]. However, cases, HIV treatment-experienced individuals, were notable for negative correlations. Thus, ART (and perhaps the HIV virus) is likely involved in the dysregulation of cholesterol biosynthesis prior to clinical and laboratory manifestations of hyperlipidemia in PLWH. HMGCR and ABCA1 could serve as biomarkers to predict the onset of cholesterol dysregulation.

Under physiologic conditions, if intracellular levels of cholesterol become low, SREBP-2 is cleaved from the endoplasmic reticulum (ER) and migrates into the nucleus [25] causing increased expression of HMGCR (the rate-limiting step in the synthesis of cholesterol) and LDLR (a membrane-associated cholesterol receptor) [26] and decreased expression of ABCA1 (a cholesterol efflux protein). These regulatory mechanisms ensure cellular homeostasis. If there is an intracellular accumulation of cholesterol, the expression of ABCA1 is increased to facilitate reverse cholesterol transport out of the cell [27]. HIV infection disrupts cholesterol efflux by ABCA1, however, there is no consensus on the effect of subsequent introduction of ART on ABCA1 expression [28, 29].

ABCA1 is a member of a superfamily of ATP-binding cassette (ABC) transporters that are involved in transporting molecules across cellular membranes. ABCA1 is involved with exporting phospholipids and cholesterol to apolipoproteins to form HDL cholesterol. Mutations in ABCA1 have been associated with low levels of HDL as observed in Tangier Disease [30, 31]. There are reports of HIV treatment-naïve individuals with upregulation of ABCA1, which normalizes upon initiation of ART [28]. Other studies have found downregulation of ABCA1 in HIV treatment naïve individuals [32]. The downregulation of ABCA1 in HIV treatment-naïve individuals has been implicated on HIV Nef protein; Nef protein downregulates ABCA1 leading to impaired efflux of cholesterol resulting in intracellular accumulation of cholesterol [33–35]. This effect is reversed with initiation of ART [36].

The mean viral load of our cases was 23 (range, 20–79) copies/mL (Table 1). Therefore, the effect of HIV via the Nef protein may not play a significant role in our cohort. Given the lack of consensus on the role of ART on ABCA1 levels and the lack of documentation of levels in patients with sustained viral suppression, we measured the level of ABCA1 expression in our cohort of patients. The mean duration of therapy among cases was 4.77 (range, 1–7.5) years. We observed an upregulation of ABCA1 gene expression in cases as compared to healthy controls. Is it plausible that the continued exposure to ART in our cohort led to upregulation of ABCA1?

It is also interesting that we found upregulation in the expression levels of HMGCR mRNA and protein levels; this is counterintuitive with upregulation of ABCA1 mRNA expression (ABCA1 protein expression tended to be high but did not reach significance (p = 0.05) as the former works to increase the intracellular cholesterol levels and the latter does the opposite. A plausible explanation is that the upregulation of HMGCR is the inciting event that results in intracellular cholesterol accumulation, especially given that the increased gene expression of ABCA1 does not reflect in protein expression. Thus, the cell, in an attempt to restore homeostasis, increases the gene expression of ABCA1. This hypothesis suggests then that if the cell is unable to cause an increase in ABCA1 protein levels, it could face the dilemma of intracellular cholesterol accumulation, a harbinger of MetS.

Another possible explanation could be that there is dysregulation of cholesterol biosynthesis. We cannot tease out this conundrum without intracellular cholesterol levels. However, the serum levels of cholesterol in cases were within normal range (based on the American Cardiology Society recommendations) implying the increase in ABCA1 mRNA expression likely predates clinically observable cholesterol perturbation (Table 2).

In the absence of intracellular cholesterol data, we performed a correlation analysis of mRNA expression of cholesterol biosynthesis genes. We observed a normal association of genes involved with cholesterol sensing—SREBP-2, HMGCR and LDLR in controls (Fig 2). In cases, there was negative correlation between SREBP-2 and HMGCR or LDLR (Fig 2). In health, SREBP-2 senses intracellular cholesterol levels and upregulates cholesterol synthesis via HMGCR and uptake from the extracellular environment via LDL receptors (LDLR) [37, 38]. Our finding suggests a dysregulation of cholesterol biosynthesis, particularly sensing by SREBP-2 in cases.

Although, our study is one of the first studies to report potential dysregulation of cholesterol biosynthesis in HIV treatment-experienced individuals, it has several limitations. First, it is a cross-sectional study and not designed to assess causality. Second, it was an exploratory pilot sub-study with small sample size to test and generate hypotheses. Third, we did not quantify intracellular cholesterol levels to assess the effect of intracellular cholesterol on the genes studied. Fourth, the effect of HIV infection itself, say through HIV Nef protein, was not assessed since we did not have access to HIV treatment-naïve individuals with higher viral loads. We were also unable to obtain the cholesterol levels of the healthy controls, instead, we compared the cholesterol levels of our cases to the upper limit of normal as published by the American Cardiology Society. Further studies are need with larger sample size and prospective design to validate our findings.

In conclusion, if our findings are validated, cholesterol biosynthesis genes could serve as biomarkers for predicting PLWH who will develop MetS and/or other lipid abnormalities and also for monitoring of treatment response of MetS and/or other lipid abnormalities in PLWH.

## Supporting information

S1 File Supplementary supporting information file. (PDF)
