# Supplementary material for: Dysregulation of sterol regulatory element-binding protein 2 gene in HIV treatment-experienced individuals
Source: PLoS One. 2019 Dec 17;14(12):e0226573. doi: 10.1371/journal.pone.0226573 (PMC6917281; doi:10.1371/journal.pone.0226573)
Supplement: S1 File — (PDF) [file pone.0226573.s001.pdf]

GENE EXPRESSION

SREBP

	Controls	Cases
1	0.698556	0.498611
3	0.2814	0.147
5	0.4122	0.2721
6	0.1481	0.0911
7	0.2095	0.2833
9	0.380547	0.317665
12	0.2064	0.18
15'	0.3406	0.2766
16	0.229	0.186
17	0.0971	0.1214
18	0.34	0.27
19	0.62	0.04
20'	0.1736	0.3132
21	0.2326	0.2071
22	0.27	0.2
23	0.1232	0.1918
24	0.1066	0.1484
25	0.1792	0.2757

HMGCR

	Controls	Cases
1	0.0445641	0.0382566
3	0.0426	0.0781
5	0.0486	0.0486
6	0.0198	0.0442
7	0.0515	0.0645
9	0.0228132	0.0214385
12	0.0689	0.0592
15'	0.0616	0.075
16	0.0256	0.0252
17	0.0106	0.0157
18	0.0342	0.0376
19	0.0562	0.1258
20'	0.0153	0.0385
21	0.0337	0.0337
22	0.0325	0.0453
23	0.0285	0.0275
24	0.0252	0.0252
25	0.0214	0.0262

LDLR

	Controls	Cases
1	0.023093	0.007633
3	0.0213	0.0227
5	0.0343	0.0385
6	0.0108	0.0259
7	0.0172	0.0674
9	0.01274	0.007585
12	0.0447	0.0224
15'	0.0566	0.022
16	0.0396	0.0288
17	0.0053	0.0102
18	0.0183	0.0203
19	0.0607	4.5342
20'	0.0105	0.0178
21	0.0345	0.0223
22	0.0309	0.0246
23	0.0127	0.0229
24	0.01	0.0151
25	0.0279	0.0169

ABCA1

	Controls	Cases
1	0.042285	0.0400593
3	0.0164	0.0174
5	0.0244	0.0279
6	0.0088	0.0331
7	0.0104	0.1038
9	0.0269224	0.0263433
12	0.0275	0.0328
15'	0.0224	0.0204
16	0.0074	0.0146
17	0.0124	0.0214
18	0.0282	0.0243
19	0.0239	0.0367
20'	0.012	0.0177
21	0.0154	0.0214
22	0.0152	0.038
23	0.0131	0.0071
24	0.0077	0.0144
25	0.0085	0.0502

	AMPKA1 Controls	Cases		AMPKB2 Controls	Cases
1	0.105921	0.110045	1	0.020833	0.016973
3	0.1898	0.2395	3	0.0629	0.0322
5	0.2192	0.2192	5	0.088	0.0592
6	0.1232	0.388	6	0.0564	0.0132
7	0.1847	0.1847	7	0.0612	0.0228
9	0.13334	0.12444	9	0.013378	0.012542
12	0.1904	0.1523	12	0.0503	0.0322
15'	0.2351	0.1554	15'	0.069	0.0326
16	0.1456	0.1341	16	0.0341	0.0345
17	0.1194	0.1538	17	0.0177	0.0289
18	0.07	0.09	18	0.02	0.03
19	0.14	0.5435	19	0.06	0.0669
20'	0.1273	0.1799	20'	0.0357	0.0593
21	0.1769	0.1769	21	0.0435	0.0713
22	0.06	0.15	22	0.03	0.03
23	0.111	0.0852	23	0.0333	0.0343
24	0.0968	0.0968	24	0.0362	0.0419
25	0.1269	0.1463	25	0.0347	0.0345

PROTEIN EXPRESSION

HMGCR Controls	Cases	ABCA1 Controls	Cases
0.013621	0.425175	1.540717	30.93929
0.09096	471.0768	0.718399	149.3821
0.026546	0.057341	0.426135	185.5044
0.012876	0.361207	49.28171	0.892158
0.002541	181.4058	0.449016	152.3385
0.00137	2.673022	0.01287	1090.835
0.004122	0.390951	0.002394	1.013694
0.158764	0.051673	0.010144	0.381224
