# Supplementary material for: Dysregulation of sterol regulatory element-binding protein 2 gene in HIV treatment-experienced individuals
Source: PLoS One. 2019 Dec 17;14(12):e0226573. doi: 10.1371/journal.pone.0226573 (PMC6917281; doi:10.1371/journal.pone.0226573)
Supplement: Attachment — Submitted filename: Response to Reviewers.docx [file pone.0226573.s002.docx]

Response to Reviewers’ comments for manuscript # PONE-D-19-26239

We are grateful to the reviewers for their insightful comments and suggestions for our manuscript # PONE-D-19-26239. We have considered the suggestions and made the recommended changes. Please find an itemized response to all the issues and criticisms. Response to comments are shown in italics.

Major concerns:
1. According to the study hypothesis, ART could influence cholesterol biosynthesis gene and dysregulation of genes could cause cholesterol abnormality or MetS, the controls were supposed to be HIV-infected individuals without receiving ART, rather than HIV-uninfected individuals. If HIV-uninfected individuals were chosen as controls, we could not tell whether the association was owing to HIV infection, ART, or other factors. Also, was the status of MetS or lipid abnormality considered in the inclusion and exclusion of participants?

*Response: Thank you for this insightful comment. The best control would have been HIV treatment-naïve individuals. With the change in ART guidelines to treat all patients irrespective of CD4 count, we don’t have that many treatment-naïve individuals in the clinic we enrolled from. Our cohort of cases had sustained viral suppression, which we used as proxy for limited contribution of HIV infection per se on our findings. However, we cannot rule out the effect of HIV infection, therefore, we will err on the safe side and attribute the effects to ART, HIV infection or both. We have expanded upon this in the second paragraph of the introduction (Lines 58 to 60) as well as in the discussion (Lines 239 to 241). Cases were individuals on ART without clinical and/or laboratory toxicities including MetS. We have clarified this in the methods’ section: Lines 83 – 85.*

2.In Table 2: 
(i)The means of cholesterol, HDL, LDL and triglycerides value in controls were missing, which were essential for analysis in the study. Authors are suggested to show that. Please add the status of MetS or lipid abnormality, and it would be better than only presenting the means of lipid-related index.

*Response: Unfortunately, we do not have the lipid profiles for the healthy controls. We report the cholesterol values of the cases and compare them to the American Cardiology Society (ACS) reference ranges. We have also reported this as one of the limitations of our study (Lines 305 -307). All the patients selected in this study, had hitherto, not been diagnosed with a lipid abnormality or signs of MetS.*

(ii)Are all the continuous variables presented in mean and range, without any one in median and interquartile? We are customary to present continuous variables following normal distribution in means±SDs; otherwise, in medians (25th-75th percentile).

*Response: We appreciate this comment. The continuous variables are now presented in medians with 25th-75th percentiles as our data is likely non-normal in distribution (please see lines 131-135). We have also added the percentiles to the text for clarity.*

(iii)Based on the statement in “Study participants and procedures”, I find participants of this study were a part of participants included in reference #17. However, the minimum age of participants in cases (age of 30) and controls (age of 32) was beyond the age range of positive controls and negative controls in reference #17 (as it was shown in the supplementary Table 1), respectively. Clarity is required on this point.

*Response: We appreciate and are grateful to the reviewer for picking up this huge discrepancy. We have corrected this discrepancy; we apologize for the error (see Table 2 and Result’s section, line 141).*

(iv)Please provide the corresponding full name of NRTI and NNRTI in the footnote.

*Response: We have provided the full names, see lines 158 to 159.*

3. Authors were supposed not to leave out the message “The difference of AMPK B2 mRNA expression among cases and controls were neither non-significant” in Line 159-161. How about LDLR and AMPK A1?

*Response: We have added a sentence about the lack of a statistically significant difference in the expression of AMPK A1 and LDLR when the respective gene expressions are compared between cases and controls (Lines 172-174).*

4. Line 162: I am confused about how to conduct stratified analysis based on “drug type”. Detailed information about this would be preferred. Considering the small total sample size, it might happen that the number of participants is too low to draw warranted conclusions, so would it be meaningful to do this analysis?

*Response: We initially did sub analyses but decided against it for this very reason. We have deleted that sentence.*

5. In “Protein expression of cholesterol biosynthesis genes in study participants” part, only participants with sufficient samples for western blot were included. My concern is about whether included participants and excluded participants were comparable in mRNA expression levels? Authors are suggested to show the result of comparison in the text.

*Response: We appreciate this insightful comment. In comparing participants with protein expression data with those without protein expression data, there was no statistically significant difference in mRNA expression, decreasing the likelihood that the cohort of patients with western blot results represents a non-random sampling. In other words, the included and excluded participants have comparable mRNA expression levels. Please see the newly attached figure (Figure 2) as well as the addendum in the Results Section, sub-section: “Protein expression of cholesterol biosynthesis genes in study participants”(lines 198 to 202) .*

6.Line 196-198: 
(i)Authors used linear regression model to examine correlation, which was inconsistent with the statement in “Statistical analysis” (in Line 133-134). If you specify Pearson or Spearman correlation as the method to be used, correlation coefficient with its 95% CI and P value should be reported.

*Response: Linear regression was indeed the method used to determine association and this has been corrected in the text (line 134).*

(ii)“significance was noted for p values ≤ 0.05” was not accurate. P<0.05 would be considered significant, whereas P=0.05 wouldn’t be.

*Response: We have adjusted our significance threshold to be P<0.05 (line 135) and throughout the entire paper.*

7.In Figure 2 (D), whether the point in upper left was outlier? Please check and explain.

*Response: It is indeed an outlier. When we excluded this outlier, the data still reflected negative correlation, which is very different than the positive correlation that is expected in non-diseased states. We have included this in the text under the section on “Correlation of cholesterol biosynthesis genes in study participants” in the results (see lines 220 to 221).*

8.Due to the fact that the study is a cross-sectional design, authors should be more cautious and not overwhelm the findings. Conclusion should be modified accordingly.

*Response: We agree with the reviewer. We have made changes throughout the manuscript to ensure that we draw appropriate conclusions based on our results and highlight inferences as hypotheses that need to be proven.*
