# Supplementary material for: Dysregulation of sterol regulatory element-binding protein 2 gene in HIV treatment-experienced individuals
Source: PLoS One. 2019 Dec 17;14(12):e0226573. doi: 10.1371/journal.pone.0226573 (PMC6917281; doi:10.1371/journal.pone.0226573)
Supplement: Peer review history

# Peer review: Decision Letter 0

**DOI:** 10.1371/journal.pone.0226573.r001

**Authors:** Ying-Mei Feng

17 Oct 2019

PONE-D-19-26239

Dysregulation of Sterol Regulatory Element-Binding Protein 2 Gene in HIV Treatment-Experienced Individuals

PLOS ONE

Dear Dr. Paintsil,

Thank you for submitting your manuscript to PLOS ONE. After careful consideration, we feel that it has merit but does not fully meet PLOS ONE’s publication criteria as it currently stands. Therefore, we invite you to submit a revised version of the manuscript that addresses the points raised during the review process.

In the cross-sectional study including 18 HIV treatment-experienced individuals (cases) and 18 HIV-uninfected individuals (controls), authors investigated the association between ART and cholesterol biosynthesis genes expression, both in mRNA and protein expression level. The results showed that mRNA and protein expressions of HMGCR and ABCA1 were significantly upregulated in cases compared to controls. Furthermore, mRNA expression of SREBP-2 was positively correlated with that of HMGCR and LDLR in controls, whereas mRNA expression of SREBP-2 was negatively correlated with that of HMGCR and LDLR in cases. This exploratory pilot study might provide some evidence for the underlying mechanisms of developing cholesterol abnormality in HIV treatment-experienced patients. 

Major concerns:

1.According to the study hypothesis, ART could influence cholesterol biosynthesis gene and dysregulation of genes could cause cholesterol abnormality or MetS, the controls were supposed to be HIV-infected individuals without receiving ART, rather than HIV-uninfected individuals. If HIV-uninfected individuals were chosen as controls, we could not tell whether the association was owing to HIV infection, ART, or other factors. Also, was the status of MetS or lipid abnormality considered in the inclusion and exclusion of participants?

2.In Table 2: 

(i)The means of cholesterol, HDL, LDL and triglycerides value in controls were missing, which were essential for analysis in the study. Authors are suggested to show that. Please add the status of MetS or lipid abnormality, and it would be better than only presenting the means of lipid-related index.

(ii)Are all the continuous variables presented in mean and range, without any one in median and interquartile? We are customary to present continuous variables following normal distribution in means±SDs; otherwise, in medians (25th-75th percentile). 

(iii)Based on the statement in “Study participants and procedures”, I find participants of this study were a part of participants included in reference #17. However, the minimum age of participants in cases (age of 30) and controls (age of 32) was beyond the age range of positive controls and negative controls in reference #17 (as it was shown in the supplementary Table 1), respectively. Clarity is required on this point. 

(iv)Please provide the corresponding full name of NRTI and NNRTI in the footnote.

3.Authors were supposed not to leave out the message“The difference of AMPK B2 mRNA expression among cases and controls were neither non-significant” in Line 159-161. How about LDLR and AMPK A1? 

4.Line 162: I am confused about how to conduct stratified analysis based on “drug type”. Detailed information about this would be preferred. Considering the small total sample size, it might happen that the number of participants is too low to draw warranted conclusions, so would it be meaningful to do this analysis?

5.In “Protein expression of cholesterol biosynthesis genes in study participants” part, only participants with sufficient samples for western blot were included. My concern is about whether included participants and excluded participants were comparable in mRNA expression levels? Authors are suggested to show the result of comparison in the text.

6.Line 196-198: 

(i)Authors used linear regression model to examine correlation, which was inconsistent with the statement in “Statistical analysis” (in Line 133-134). If you specify Pearson or Spearman correlation as the method to be used, correlation coefficient with its 95% CI and P value should be reported.

(ii)“significance was noted for p values ≤ 0.05” was not accurate. P<0.05 would be considered significant, whereas P=0.05 wouldn’t be.

7.In Figure 2 (D), whether the point in upper left was outlier? Please check and explain.

8.Due to the fact that the study is a cross-sectional design, authors should be more cautious and not overwhelm the findings. Conclusion should be modified accordingly.

We would appreciate receiving your revised manuscript by Dec 01 2019 11:59PM. When you are ready to submit your revision, log on to https://www.editorialmanager.com/pone/ and select the 'Submissions Needing Revision' folder to locate your manuscript file.

If you would like to make changes to your financial disclosure, please include your updated statement in your cover letter.

To enhance the reproducibility of your results, we recommend that if applicable you deposit your laboratory protocols in protocols.io, where a protocol can be assigned its own identifier (DOI) such that it can be cited independently in the future. For instructions see: http://journals.plos.org/plosone/s/submission-guidelines#loc-laboratory-protocols

Please include the following items when submitting your revised manuscript:

- A rebuttal letter that responds to each point raised by the academic editor and reviewer(s). This letter should be uploaded as separate file and labeled 'Response to Reviewers'.
- A marked-up copy of your manuscript that highlights changes made to the original version. This file should be uploaded as separate file and labeled 'Revised Manuscript with Track Changes'.
- An unmarked version of your revised paper without tracked changes. This file should be uploaded as separate file and labeled 'Manuscript'.

Please note while forming your response, if your article is accepted, you may have the opportunity to make the peer review history publicly available. The record will include editor decision letters (with reviews) and your responses to reviewer comments. If eligible, we will contact you to opt in or out.

We look forward to receiving your revised manuscript.

Kind regards,

Ying-Mei Feng

Academic Editor

PLOS ONE

Journal requirements:

When submitting your revision, we need you to address these additional requirements.

1. Please ensure that your manuscript meets PLOS ONE's style requirements, including those for file naming. The PLOS ONE style templates can be found at http://www.journals.plos.org/plosone/s/file?id=wjVg/PLOSOne_formatting_sample_main_body.pdf and http://www.journals.plos.org/plosone/s/file?id=ba62/PLOSOne_formatting_sample_title_authors_affiliations.pdf

# Author comment: Author response to Decision Letter 0

**DOI:** 10.1371/journal.pone.0226573.r002

12 Nov 2019

Response to Reviewers’ comments for manuscript # PONE-D-19-26239

We are grateful to the reviewers for their insightful comments and suggestions for our manuscript # PONE-D-19-26239. We have considered the suggestions and made the recommended changes. Please find an itemized response to all the issues and criticisms. Response to comments are shown in italics.

Major concerns:

1. According to the study hypothesis, ART could influence cholesterol biosynthesis gene and dysregulation of genes could cause cholesterol abnormality or MetS, the controls were supposed to be HIV-infected individuals without receiving ART, rather than HIV-uninfected individuals. If HIV-uninfected individuals were chosen as controls, we could not tell whether the association was owing to HIV infection, ART, or other factors. Also, was the status of MetS or lipid abnormality considered in the inclusion and exclusion of participants?

Response: Thank you for this insightful comment. The best control would have been HIV treatment-naïve individuals. With the change in ART guidelines to treat all patients irrespective of CD4 count, we don’t have that many treatment-naïve individuals in the clinic we enrolled from. Our cohort of cases had sustained viral suppression, which we used as proxy for limited contribution of HIV infection per se on our findings. However, we cannot rule out the effect of HIV infection, therefore, we will err on the safe side and attribute the effects to ART, HIV infection or both. We have expanded upon this in the second paragraph of the introduction (Lines 58 to 60) as well as in the discussion (Lines 239 to 241). Cases were individuals on ART without clinical and/or laboratory toxicities including MetS. We have clarified this in the methods’ section: Lines 83 – 85.

2.In Table 2: 

(i)The means of cholesterol, HDL, LDL and triglycerides value in controls were missing, which were essential for analysis in the study. Authors are suggested to show that. Please add the status of MetS or lipid abnormality, and it would be better than only presenting the means of lipid-related index.

Response: Unfortunately, we do not have the lipid profiles for the healthy controls. We report the cholesterol values of the cases and compare them to the American Cardiology Society (ACS) reference ranges. We have also reported this as one of the limitations of our study (Lines 305 -307). All the patients selected in this study, had hitherto, not been diagnosed with a lipid abnormality or signs of MetS. 

(ii)Are all the continuous variables presented in mean and range, without any one in median and interquartile? We are customary to present continuous variables following normal distribution in means±SDs; otherwise, in medians (25th-75th percentile). 

Response: We appreciate this comment. The continuous variables are now presented in medians with 25th-75th percentiles as our data is likely non-normal in distribution (please see lines 131-135). We have also added the percentiles to the text for clarity.

(iii)Based on the statement in “Study participants and procedures”, I find participants of this study were a part of participants included in reference #17. However, the minimum age of participants in cases (age of 30) and controls (age of 32) was beyond the age range of positive controls and negative controls in reference #17 (as it was shown in the supplementary Table 1), respectively. Clarity is required on this point. 

Response: We appreciate and are grateful to the reviewer for picking up this huge discrepancy. We have corrected this discrepancy; we apologize for the error (see Table 2 and Result’s section, line 141).

(iv)Please provide the corresponding full name of NRTI and NNRTI in the footnote.

Response: We have provided the full names, see lines 158 to 159.

3. Authors were supposed not to leave out the message “The difference of AMPK B2 mRNA expression among cases and controls were neither non-significant” in Line 159-161. How about LDLR and AMPK A1? 

Response: We have added a sentence about the lack of a statistically significant difference in the expression of AMPK A1 and LDLR when the respective gene expressions are compared between cases and controls (Lines 172-174).

4. Line 162: I am confused about how to conduct stratified analysis based on “drug type”. Detailed information about this would be preferred. Considering the small total sample size, it might happen that the number of participants is too low to draw warranted conclusions, so would it be meaningful to do this analysis?

Response: We initially did sub analyses but decided against it for this very reason. We have deleted that sentence. 

5. In “Protein expression of cholesterol biosynthesis genes in study participants” part, only participants with sufficient samples for western blot were included. My concern is about whether included participants and excluded participants were comparable in mRNA expression levels? Authors are suggested to show the result of comparison in the text.

Response: We appreciate this insightful comment. In comparing participants with protein expression data with those without protein expression data, there was no statistically significant difference in mRNA expression, decreasing the likelihood that the cohort of patients with western blot results represents a non-random sampling. In other words, the included and excluded participants have comparable mRNA expression levels. Please see the newly attached figure (Figure 2) as well as the addendum in the Results Section, sub-section: “Protein expression of cholesterol biosynthesis genes in study participants”(lines 198 to 202) .

6.Line 196-198: 

(i)Authors used linear regression model to examine correlation, which was inconsistent with the statement in “Statistical analysis” (in Line 133-134). If you specify Pearson or Spearman correlation as the method to be used, correlation coefficient with its 95% CI and P value should be reported.

Response: Linear regression was indeed the method used to determine association and this has been corrected in the text (line 134).

(ii)“significance was noted for p values ≤ 0.05” was not accurate. P<0.05 would be considered significant, whereas P=0.05 wouldn’t be.

Response: We have adjusted our significance threshold to be P<0.05 (line 135) and throughout the entire paper.

7.In Figure 2 (D), whether the point in upper left was outlier? Please check and explain.

Response: It is indeed an outlier. When we excluded this outlier, the data still reflected negative correlation, which is very different than the positive correlation that is expected in non-diseased states. We have included this in the text under the section on “Correlation of cholesterol biosynthesis genes in study participants” in the results (see lines 220 to 221).

8.Due to the fact that the study is a cross-sectional design, authors should be more cautious and not overwhelm the findings. Conclusion should be modified accordingly.

Response: We agree with the reviewer. We have made changes throughout the manuscript to ensure that we draw appropriate conclusions based on our results and highlight inferences as hypotheses that need to be proven.

Attachment Submitted filename: Response to Reviewers.docx Click here for additional data file.

# Peer review: Decision Letter 1

**DOI:** 10.1371/journal.pone.0226573.r003

**Authors:** Ying-Mei Feng

3 Dec 2019

Dysregulation of Sterol Regulatory Element-Binding Protein 2 Gene in HIV Treatment-Experienced Individuals

PONE-D-19-26239R1

Dear Dr. Paintsil,

We are pleased to inform you that your manuscript has been judged scientifically suitable for publication and will be formally accepted for publication once it complies with all outstanding technical requirements.

Within one week, you will receive an e-mail containing information on the amendments required prior to publication. When all required modifications have been addressed, you will receive a formal acceptance letter and your manuscript will proceed to our production department and be scheduled for publication.

Shortly after the formal acceptance letter is sent, an invoice for payment will follow. To ensure an efficient production and billing process, please log into Editorial Manager at https://www.editorialmanager.com/pone/, click the "Update My Information" link at the top of the page, and update your user information. If you have any billing related questions, please contact our Author Billing department directly at authorbilling@plos.org.

If your institution or institutions have a press office, please notify them about your upcoming paper to enable them to help maximize its impact. If they will be preparing press materials for this manuscript, you must inform our press team as soon as possible and no later than 48 hours after receiving the formal acceptance. Your manuscript will remain under strict press embargo until 2 pm Eastern Time on the date of publication. For more information, please contact onepress@plos.org.

With kind regards,

Ying-Mei Feng

Academic Editor

PLOS ONE

# Editor report: Acceptance letter

**DOI:** 10.1371/journal.pone.0226573.r004

**Authors:** Ying-Mei Feng

9 Dec 2019

PONE-D-19-26239R1 

Dysregulation of Sterol Regulatory Element-Binding Protein 2 Gene in HIV Treatment-Experienced Individuals 

Dear Dr. Paintsil:

I am pleased to inform you that your manuscript has been deemed suitable for publication in PLOS ONE. Congratulations! Your manuscript is now with our production department. 

If your institution or institutions have a press office, please notify them about your upcoming paper at this point, to enable them to help maximize its impact. If they will be preparing press materials for this manuscript, please inform our press team within the next 48 hours. Your manuscript will remain under strict press embargo until 2 pm Eastern Time on the date of publication. For more information please contact onepress@plos.org.

For any other questions or concerns, please email plosone@plos.org. 

Thank you for submitting your work to PLOS ONE.

With kind regards,

PLOS ONE Editorial Office Staff

on behalf of

Dr Ying-Mei Feng 

Academic Editor

PLOS ONE
